# Non-Isothermal Crystallization Kinetics of Polyamide 6/Graphene Nanoplatelets Nanocomposites Obtained via In Situ Polymerization: Effect of Nanofiller Size

**DOI:** 10.3390/polym15204109

**Published:** 2023-10-17

**Authors:** Joana Lagarinhos, Sara Magalhães da Silva, José Martinho Oliveira

**Affiliations:** 1 EMaRT Group—Emerging: Materials, Research, Technology, University of Aveiro, 3810-193 Aveiro, Portugal; joanalagarinhos@ua.pt (J.L.); martinho@ua.pt (J.M.O.); 2 School of Design, Management and Production Technologies, University of Aveiro, Estrada do Cercal 449, 3720-509 Oliveira de Azeméis, Portugal; 3 CICECO—Aveiro Institute of Materials, University of Aveiro, Campus Universitário de Santiago, 3810-193 Aveiro, Portugal

**Keywords:** Polyamide 6 (PA6), anionic ring-opening polymerization (AROP), thermoplastic resin transfer molding (T-RTM), graphene nanoplatelets (GNP), nanocomposites, non-isothermal crystallization kinetics

## Abstract

Thermoplastic resin transfer molding (T-RTM) technology was applied to synthesize graphene nanoplatelets-based nanocomposites via anionic ring-opening polymerization (AROP). Polyamide 6 (PA6) was obtained by AROP and was used as the polymeric matrix of the developed nanocomposites. The non-isothermal crystallization behavior of PA6 and nanocomposites was analyzed by differential scanning calorimetry (DSC). Nanocomposites with 0.5 wt.% of graphene nanoplatelets (GNPs) with two different diameter sizes were prepared. Results have shown that the crystallization temperature shifted to higher values in the presence of GNPs. This behavior is more noticeable for the nanocomposite prepared with smaller GNPs (PA6/GN). The crystallization kinetic behavior of all samples was assessed by Avrami and Liu’s models. It was observed that GNPs increased the crystallization rate, thus revealing a nucleating ability, and also validated the reduction of half-time crystallization values. Such tendency was also supported by the lower activation energy values determined by Friedman’s method.

## 1. Introduction

Polyamide 6 (PA6) is a thermoplastic material of particular interest in automotive applications, mainly in semi-structural parts, due to its excellent impact resistance, good strength properties and high resistance to most solvents and acids [1,2]. PA6 has emerged as an alternative to traditional automotive metal parts, capable of combining their unique mechanical properties with their lightness [3,4,5]. PA6 can be produced by hydrolytic polymerization, due to its industrial viability, controllability, and polymerization stability for large-scale operation. However, this polymerization involves various steps [1,6]. On the other hand, an anionic polymerization involves an activated monomer mechanism, where PA6 is obtained from the corresponding lactams [7,8]. The lactam polymerization can be an anionic reaction mechanism (initiated by a base) or a cationic reaction mechanism (initiated by an acid). However, cationic mechanisms are limited due to low conversions and the low molar masses of the final products [8,9].

The anionic ring-opening polymerization (AROP) of ε-caprolactam (CL) into PA6 is among one of the most developed forms of reactive processing of thermoplastics. It is based on a polymerization mechanism in which the ring-shaped (cyclic) molecules are opened, giving rise to linear monomers or oligomers [8,9]. AROP is the fastest process for PA6 production; it is characterized by short polymerization times, resulting in a faster cycle time, and, consequently, a more efficient production (compared to thermosets). In addition to the monomer, a catalyst, and an activator are needed to initiate and maintain the reaction [10]. This type of polymerization has emerged as a clean alternative to polymerization routes as it does not require hazardous solvents [1,11].

Thermoplastic resin transfer molding (T-RTM) is a technology able to produce a thermoplastic material by combining a precursor with low viscosity, a catalyst, and an activator [12,13,14]. The reactive mixture is injected into a mold and polymerizes inside it (in situ). Ensuring appropriate processing conditions for polymerization in situ, namely, adequate temperature, polymerization time, and inert atmosphere, is necessary [3]. PA6 can be synthesized by AROP using the T-RTM technology, due to the characteristic low viscosity of ε-caprolactam combined with a fast polymerization rate [7,8,9]. PA6 synthesized by AROP was found to produce high molecular weights, which results in tougher materials that are adequate for industrial applications [15]. A superior mechanical performance was also observed, such as impact resistance, abrasion resistance, and strength properties, which were above those normally found in melt-processed PA6 [3,13].

The diffusion of thermoplastics has aroused high interest in the development of composite materials. Thermoplastics reveal high impact resistance, short processing time, and recyclability [4,16,17]. Recently, for structural applications, several reinforcing fabrics, such as carbon [3,18] and glass fibers [19], aramid [20], attapulgite [21,22], or carbon nanotubes (CNTs) [23,24], are being used to reinforce thermoplastic composites. The incorporation of a reinforcement phase into a polymeric matrix to create a composite material results in significant improvements over unfilled polymers [25,26]. These improvements include enhanced mechanical properties, increased thermal stability, and improved electrical properties, all of which can be achieved with relatively low filler content [27,28]. In the case of using PA6 as the polymeric matrix, the low viscosity of PA6 precursors enabled the reactive processing of thermoplastic composites by T-RTM. This has opened the door for new applications and materials development where thermosets have traditionally been used [29,30]. An example of such development is the study of graphene nanoplatelets (GNPs) as a cost-effective filler in PA6-based nanocomposites [31,32]. GNPs exhibit improved mechanical and thermal properties such as high Young’s modulus, high fracture strength and thermal conductivity [33,34,35]. Due to their promising properties, GNPs-based nanocomposites have been used in a wide range of applications, including automotives, electronics, packaging, aerospace, military, buildings, and construction [27,36,37]. GNPs offer an appealing prospect due to their unique properties and the abundance of their precursor, graphite. In addition, the straightforward and cost-effective physicochemical methods employed in the production of GNPs can further contribute to their viability [35,38].

Several studies have shown that the addition of nanofillers can influence properties, such as toughness, and impact performance, thermal stability, and electrical and thermal conductivity [39]. The study of crystallization behavior is, therefore, an important tool for optimizing processing conditions, contributing to shorter industrial cycles and reducing manufacturing costs. This trend was observed by Fu, X. et al. [40], who have studied the effect of multilayer graphene (MG) content of 0.01–0.5 wt.% on PA6. According to the authors, the addition of MG affected the crystallinity degree, which varied within a small range between 32.8 and 34.8%. This result was higher than that of the PA6 matrix (31.8%), indicating that MG can promote crystallization by acting as a nucleating agent. In addition, the crystallization peaks of composites become narrower compared to PA6, which means that MG loading can also increase the crystallization rate of PA6-based nanocomposites. Yang Chen and co-workers [41], prepared exfoliated graphite-filled PA6 composites and evaluated the influence of graphite (0–20 wt.%) on the thermal properties of the composites. The authors showed that all PA6/graphite composites had higher crystallization rates (varying from 2.97–5.08 min) than pure PA6 (6.28 min). However, the addition of 20 wt.% graphite slowed down the crystallization rate, as MG could induce a physical barrier and reduce the mobility of polymer chains. This behavior indicated the nucleating ability of graphite, which provided nucleation sites and facilitated the crystallization process when small amounts of graphite are used, while higher concentrations of graphite may hinder the growth of crystallites. Nevertheless, none of these studies are related to the study of non-isothermal crystallization behavior and kinetics of PA6-based nanocomposites prepared by T-RTM via AROP.

This work aims to investigate the effect of GNPs, with different diameters, on the crystallization behavior of PA6 under non-isothermal conditions prepared by T-RTM technology. The crystallization was conducted at non-isothermal conditions to simulate the T-RTM process. It will provide significant information about the polymerization mechanisms and kinetics of PA6/GNPs to guarantee parts with dimensional accuracy and reduced defects, such as warping [15,42,43]. Kinetic studies using Avrami [44,45] and Liu [46] models were carried out to evaluate the influence of GNPs in the crystallization kinetics of PA6. The crystallization activation energy (E_c_) was calculated using Friedman’s methodology [47]. In addition, the morphology of GNPs was observed using scanning electron microscopy (SEM), and the dispersion and distribution of the GNPs into PA6 was evaluated via polarized optical microscopy (POM).

## 2. Materials and Methods

### 2.1. Materials

PA6 was prepared by T-RTM technology using the following materials: (1) monomer: ε-caprolactam (CL), AP-Nylon^®^; (2) catalyst: Bruggolen^®^ C10; and (3) activator: Bruggolen^®^ C20P. All chemical components were purchased from L. Brüggemann GmbH and Co. KG, Heilbronn, Germany. The GNP fillers were supplied by NanoXplore (Saint-Laurent, QC, Canada) and two different sizes were considered, namely, D90 < 50 μm (defined as GN) and D90 < 70 μm (defined as GP). The bulk density of all GNP fillers is reported to be 0.2–0.3 g/cm^3^ (data from technical datasheets).

### 2.2. PA6 and PA6/GNPs Nanocomposites Preparation

PA6 was prepared using the same compositions and T-RTM laboratory system as described in a previous study [48]. The nitrogen pressure (3 bar) and vacuum conditions (150 mbar) were carefully adjusted to reduce the existence of voids and to ensure the integrity of the manufactured parts. PA6/GNPs nanocomposites were prepared by pre-dispersing 0.5 wt.% GN (PA6/GN) and GP (PA6/GP) in molten CL using a Hielscher ultrasonic device UP 200 S (200 watts, frequency 24 kHz) to achieve better dispersion of GNPs in the PA6 matrix. The sonication time was set to 20 min for GN and 25 min for GP. Then, the catalyst and activator were added, and the polymerization process proceeded as previously described [48]. The selection of 0.5 wt.% as GNPs concentration was previously investigated, where the influence of different concentrations of GNPs on the final properties of the PA6 matrix were evaluated [49].

### 2.3. Thermal Analyses

The thermal properties and the non-isothermal crystallization behavior and kinetics of PA6 and PA6/GNPs were studied with differential scanning calorimetry (DSC). A Shimadzu DSC-60 under air atmosphere was used. The thermal history of the samples was erased by heating from room temperature to 250 °C at 20 °C/min and remained at this temperature for 2 min. Non-isothermal crystallization behavior was investigated by cooling the samples from 250 °C to −20 °C at various cooling rates of 5, 10, 15 and 20 °C/min. This cycle was duplicated, and only the second run was used to evaluate the crystallization behavior. Crystallization temperature (T_c_), crystallization enthalpy (ΔH_c_), and melting temperature (T_m_) were determined from the DSC thermograms. At least three measurements were made for each testing condition.

### 2.4. Morphological Analyses

Morphological analyses were conducted to analyze the microstructure of GNPs. An SEM Hitachi S4100 was used with an acceleration voltage of 15 kV. The GNPs were assembled on a conducting carbon tape, and then the samples were sputter coated with AU/Pd for 3 min to enhance the image resolution and to prevent electrostatic charging. To evaluate the distribution of GNPs into the PA6 matrix, a Nikon Eclipse L150 microscope equipped with a digital camera (Canon 100D) was used. The analyzed samples were obtained from the longitudinal section of the specimens. Both samples were melted between two glass slides at 230 °C for 5 min to obtain thin films and were kept at this temperature for 10 min.

## 3. Results

### 3.1. GNPs and Nanocomposites Morphology

**Figure 1:**
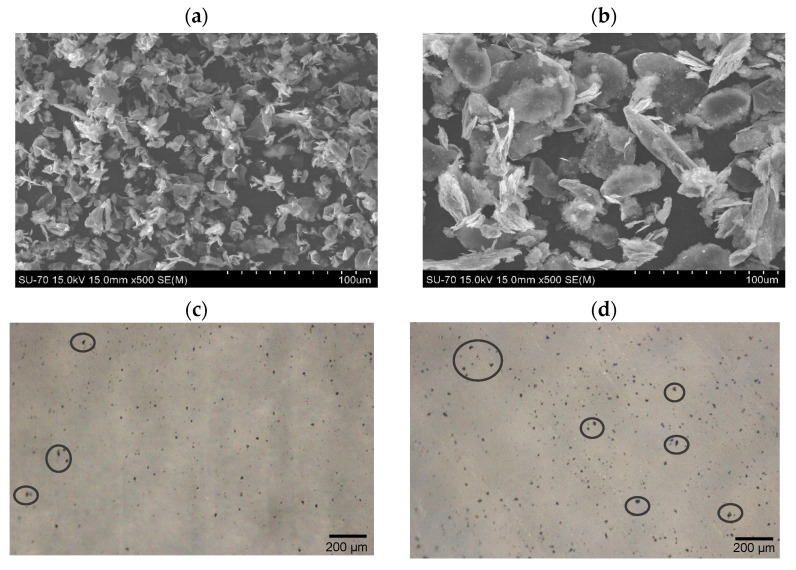
SEM images of (**a**) GN; and (**b**) GP; and optical micrographs of (**c**) PA6/GN and (**d**) PA6/GP nanocomposites (black circles highlight GNPs agglomerates).

Figure 1a,b show the morphologies of the GNPs used, under identical magnification for accurate comparison of the GNPs size. Both GNPs consist of platelet-shaped graphene layers stacked and folded together, with irregular morphology and opaque structure. The images also show that the diameter of each of the GNPs corresponds to the average diameter reported by the manufacturer.

According to the technical data sheet of the GNPs, functional groups such as carboxyl or hydroxyl groups are present at the edges of the nanoplatelets, which facilitates the distribution of GNPs in the PA6 matrix. Optical micrographs of PA6/GNPs after 10 min at 230 °C in a hot plate are shown in Figure 1c,d. Each type of GNPs was dispersed in the PA6 matrix, and it is possible to perceive the differences between the diameters of the two GNPs used in this work. A good dispersion was observed for both nanocomposites, although some agglomerates can be noticed in Figure 1d due to the larger size of GP. The nanocomposite PA6/GN shows better dispersion and less agglomeration of nanoplatelets than PA6/GP. Based on this observation, it can be assumed that a larger GNPs diameter makes the dispersion difficult and promotes the formation of aggregates. Thus, GNPs particle size can influence the mechanical properties, as reported previously [49], and the thermal properties of nanocomposites [50].

### 3.2. Non-Isothermal Crystallization Behavior of PA6 and PA6/GNPs Nanocomposites

**Figure 2:**
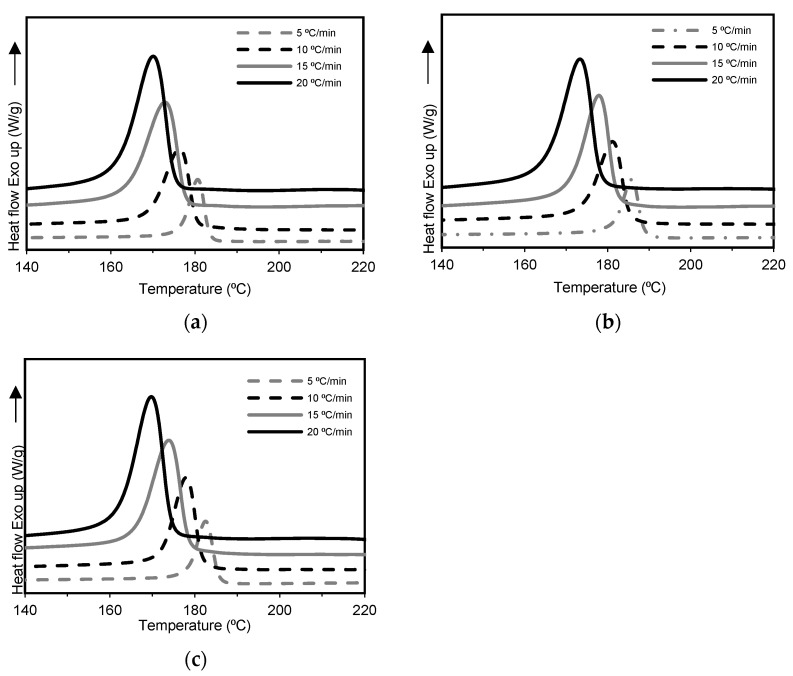
DSC curves at different cooling rates for (**a**) PA6, (**b**) PA6/GN, and (**c**) PA6/GP.

DSC measurements were conducted to investigate the effect of each GNPs type on the non-isothermal crystallization behavior of the prepared nanocomposites. The exothermic curves of PA6, PA6/GN and PA6/GP at different cooling rates are shown in Figure 2. The resulting thermal parameters are listed in Table 1.

As can be seen from Table 1, the T_m_ of PA6 and its nanocomposites were unaffected by the selected cooling rate of samples. The cooling curves (Figure 2) show that the lowest cooling rates, 5 and 10 °C/min, induce a rather narrow T_c_, whereas the highest cooling rates, 15 and 20 °C/min, induce broad crystallization curves. As expected, as the cooling rate increases, the curves, and thus the peak temperatures, shift towards lower temperatures where there is insufficient time to activate the nuclei at higher temperatures. PA6 and its nanocomposites showed a significant decrease in T_c_ of about 14 °C and 13 °C, respectively. The T_c_ values, for a given cooling rate, were higher for nanocomposites than for PA6. This behavior can indicate that PA6 crystallized earlier in the presence of GNPs, revealing a nucleation effect of GNPs on the polymeric matrix [51]. In particular, PA6/GN nanocomposites, with smaller GNPs, showed higher T_c_ values than PA6/GP. It can be assumed that lower surface area induces fewer interactions between GNPs and PA6, which makes the regular packing of PA6 polymer chains difficult [52].

The X_c_ was assessed to better understand the effect of GNPs on PA6 crystal formation. This parameter was calculated according to Equation (1):

(1) $$Xc\;\left(\%\right)=\frac{∆H_{c}}{\left(1-\alpha \right)∆H_{m}^{0}}\times 100$$

where ∆H_c_ was obtained by integrating the crystallization peak, $∆H_{m}^{0}$ corresponds to the 100% crystalline form of PA6 (which is assumed to be 190 J/g) [53], and α is the filler mass fraction. It was observed that the addition of GNPs did not significantly affect the X_c_ values. The same behavior was also previously reported [54,55,56]. Theoretically, the addition of GNPs to PA6 can have two significant effects: heterogeneous nucleation, where GNPs provide sites for polymer molecules to form crystalline structures; and, physical hindrance, where GNPs can obstruct the movement of polymer molecules [57]. As a result, the process of crystal growth can be slowed down. In the literature, studies have reported that carbon-based fillers can reduce X_c_ [58,59], but it has also been found in other studies that an increase in X_c_ was detected [60,61,62]. In this case, the addition of GNPs showed no significant changes in X_c_. The 2D structure of GNPs with a small thickness and high aspect ratio could be a critical reason for its inhibitory effect. This structural uniqueness can interfere with the molecular movement and crystalline growth of the PA6 matrix in an irregular manner.

**Figure 3:**
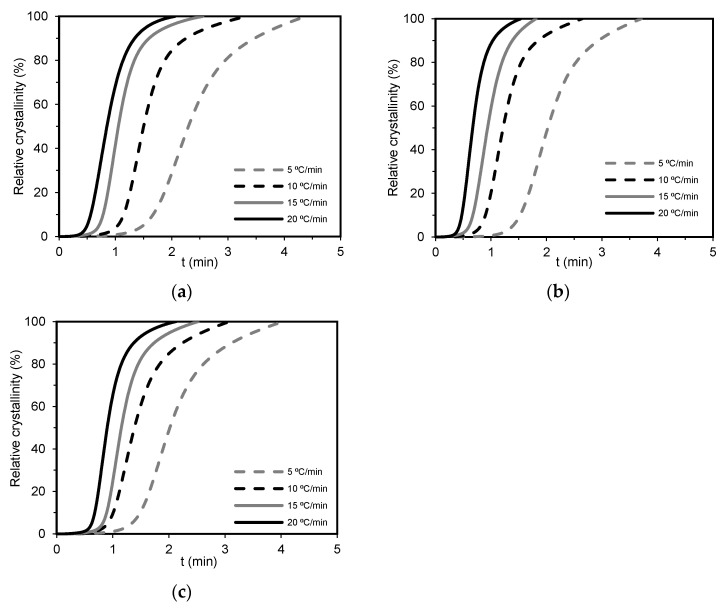
Curves of X_t_ vs. *t* under different cooling rates for (**a**) PA6, (**b**) PA6/GN, and (**c**) PA6/GP.

From the DSC thermograms, the evolution of the relative crystallinity degree (X_t_), at a certain time (*t*) can be determined by Equation (2):

(2) $$X_{t}=\frac{\int_{T_{0}}^{T}\left(\frac{dH_{c}}{dT}\right)dT}{\int_{T_{0}}^{T_{\infty }}\left(\frac{{dH}_{c}}{dT}\right)dT}$$

where T_0_ and T_∞_ are the temperatures at which crystallization begins and ends, respectively. In non-isothermal crystallization, the temperature (T) can be converted to the crystallization time [63], *t*, using the following equation:

(3) $$t=\frac{T_{0}-T}{\varphi }$$

where T is the temperature at crystallization time and φ corresponds to the cooling rate. Thus, from Equation (3), it is possible to convert X_t_ = f(T) curves from non-isothermal DSC crystallization data into X_t_ = *f*(t) curves. Figure 3 displays the X_t_ vs. *t* curves for all samples. All curves exhibited a sigmoidal shape with two non-linear parts. The early stage (the first non-linear part) corresponds to fast primary crystallization, attributed to the formation of nuclei; while the later stage (the second non-linear part) is described by a slow secondary crystallization, ascribed to the spherulitic impingement of the crystallization. The crystallization process starts at higher temperatures for slower cooling rates (5 °C/min), and it occurs for a longer time. Although the crystallization process begins at lower temperatures at higher cooling rates (20 °C/min), the duration is shorter. From these plots, the strong dependence of nucleation and growth processes on the cooling rate can be seen. When the cooling rate increases, the curves shift to the left position, indicating a faster crystallization rate. Other studies also have reported a similar trend [64,65,66].

### 3.3. Non-Isothermal Crystallization Behavior of PA6 and PA6/GNPs Nanocomposites

#### 3.3.1. Avrami Model

The Avrami [44] equation has been commonly applied to analyze the non-isothermal kinetics of the nucleation and growth phases of polymers at a fixed crystallization temperature:

(4) $$X_{t}=1-\exp(-Z_{t}t^{n})$$

where Z_t_ is the growth rate constant that includes both nucleation and growth rate, and *n* is the Avrami exponent, which depends on the shape of the crystalline units and the nucleation process. It should be noted that these parameters affect the rates of both nucleation and spherulite growth caused by their temperature dependence. The linearized form of Equation (4) can be written as follows:

(5) $$\log\left[-ln(1-X_{t})\right]=\ln\left(Zt\right)+nln\left(t\right)$$

**Figure 4:**
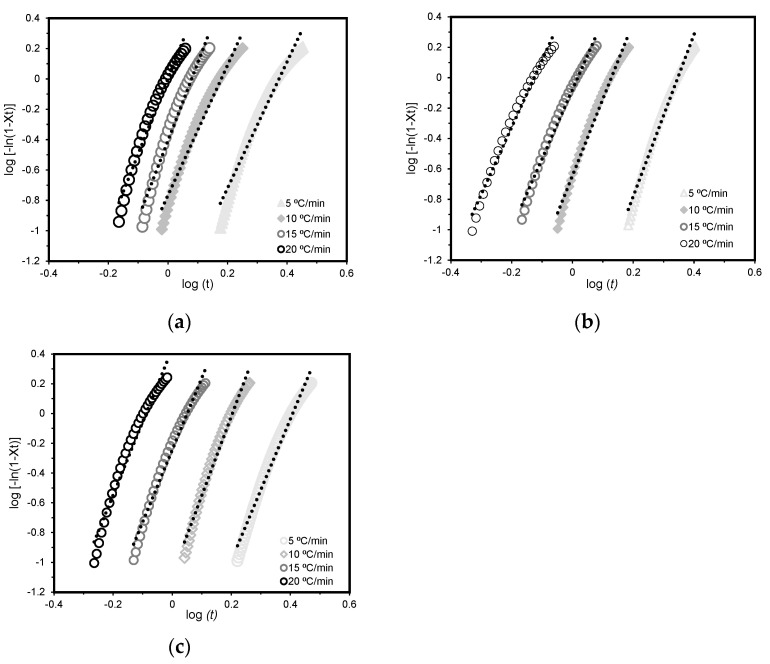
Avrami plots for (**a**) PA6, (**b**) PA6/GN, and (**c**) PA6/GP.

Figure 4 shows the curves of $\log[-\ln\left(1-X_{t})\right]$ vs. log (t) for PA6, PA6/GN, and PA6/GP at different cooling rates. The Avrami exponent *n* and the parameter rate Z_t_ can be calculated from the slope and the interception of the lines, respectively. The Avrami approach can reveal several fundamental aspects related to the crystallization mechanisms but is not able to properly describe the non-isothermal crystallization of the process, as a constant cooling rate can affect the parameters. Jeziorny et al. [67] adapted the adjustments to the Avrami model, by replacing the rate parameter Z_t_ with Z_c_ to describe the non-isothermal kinetics:

(6) $${lnZ}_{c}=\frac{\ln\left(Z_{t}\right)}{\varphi }$$

This correction is widely used to adjust Avrami’s theory to non-isothermal conditions. Avrami plots and kinetic parameters are show in Figure 4 and Table 2, respectively. X_t_ values between 10 and 80% were considered.

The data presented in Table 2 show that the presence of GNPs did not affect the crystallization kinetics of PA6. The variation of the Avrami parameter *n* with cooling rate revealed the presence of mixed nucleation and growth mechanisms. A crystallization process with *n* varying from one and two follows a one-dimensional crystal growth, and a value between two and three is a two-dimensional growth. When the *n* value is between three and four, the crystallization follows a three-dimensional (3D) growth, and for an *n* value higher than four, a complex multi-dimensional crystallization process is developed [68]. *n* values higher than three can be associated with developing a 3D growth crystallization process, implying that different growth mechanisms may occur simultaneously during the crystallization process [21]. Similar studies have reported analogous behavior [66,69,70]. Comparing overall values of PA6 and PA6/GNPs, it can be observed that marginal changes were noted at 0.5 wt.% GNPs incorporation. Melo et al. [64] also observed similar behavior when using the same amount of graphene oxide (GO) concentration. The authors reported that no significant changes in the nucleation rate were observed for 0.1 and 0.5 wt.% nanocomposites. However, when incorporating 1 and 5 wt.% GO, higher *n* values were observed with increasing cooling rates. This behavior suggests a change in the nucleation mechanism of the sample at higher nanofiller concentrations. On the other hand, increased amounts of GO also caused an increase in Z_c_ values and an increase in t_1/2_, indicating a slower crystallization rate for the nanocomposites due to the reduced mobility of the polymer chains in the presence of GO sheets.

The time to reach 50% of X_t_ is defined as the half-time crystallization (t_1/2_). This parameter is also an indicator of the crystallization rate and can be determined by Equation (7):

(7) t12=ln⁡2Zc1n

The t_1/2_ values are also given in Table 2. It can be seen that the addition of GNPs leads to a decrease in the t_1/2_ values, indicating that the addition of GNPs accelerated the crystallization rate. This can be an indication of the heterogeneous nucleation ability of GNPs [71]. In the case of nanocomposites, PA6/GN achieved lower t_1/2_ than PA6/GP. This can be due to the smaller diameter of GN, which provided more nuclei, thus promoting crystallization.

#### 3.3.2. Lui Model

The Liu [46] model was also applied to adjust the non-isothermal kinetic behavior of PA6 and nanocomposites. It was developed as an alternative method by combining the Avrami and Ozawa equations. From that, a new kinetic equation was obtained:

(8) $$logK\left(T\right)-mlog\left(\varphi \right)=logZ_{t}+nlog\left(t\right)$$

**Figure 5:**
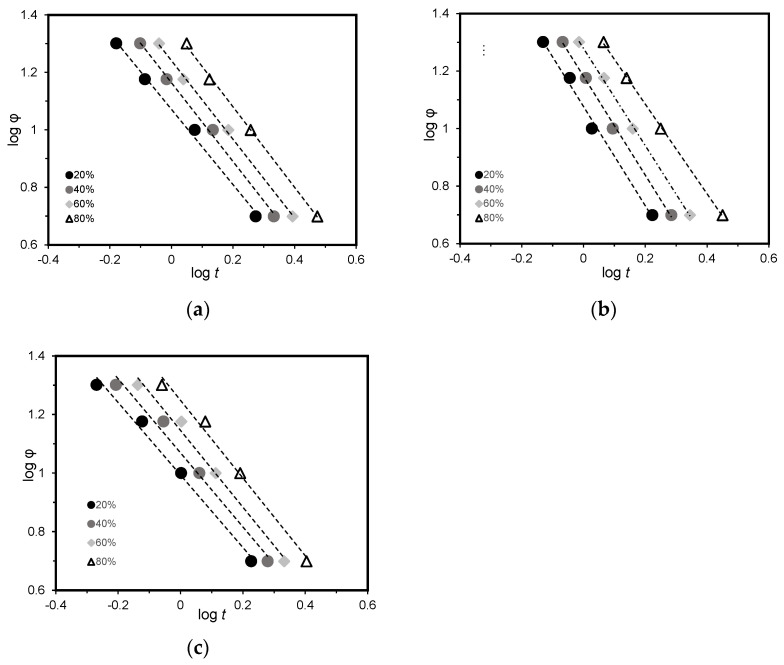
Liu plots for (**a**) PA6, (**b**) PA6/GN, and (**c**) PA6/GP.

Simplifying this equation:

(9) $$log\left(\varphi \right)=\log\left[F\left(T\right)\right]-b\;log(t)$$

where F(T) refers to the cooling rate required to reach X_t_ in a specific *t*, and *b* is the ratio between the exponents of Avrami (*n*) and Ozawa (*m*). According to Equation (9), the kinetic parameters F(T) and the b values can be determined from the intercept and the slope of the lines by plotting log φ against log t, respectively. Figure 5 shows the Liu curves for PA6 and PA6/GNPs nanocomposites, and Table 3 also presents the Liu kinetic parameters.

Parallel straight lines and linearity were attained for different X_t_, indicating that the Liu analysis can successfully describe PA6 and PA6/GNPs kinetic behavior. For a given sample, F(T) increased with increasing X_t_. Since F(T) is described as a cooling rate value, a decrease in F(T) indicates that a higher crystallization rate is required to reach a given crystallinity degree at a set time [72]. Comparing the F(T) values of PA6 with those of PA6/GNPs samples, the nanocomposites presented lower values than the pure matrix. The presence of GNPs increases the crystallization rate of the polymer and accelerates the process, implying that the GNPs facilitate the crystallization process, also demonstrating their nucleation ability [73]. Furthermore, when comparing PA6/GN with PA6/GP, it can be seen that for PA6/GP, F(T) values are higher due to the filler diameter size. At larger sizes, the interparticle distance decreases and GNPs are unable to disperse into the PA6 matrix due to the resulting aggregation from weak intermolecular forces between them [72]. The kinetic parameter *b* is almost constant for each material at different X_t_ values, ranging from 1.31 and 1.59. These values imply that significant secondary crystal growth accompanies the primary crystallization during non-isothermal crystallization. The nucleation mechanism and crystal growth geometries are similar, indicating that the method used could describe the non-isothermal crystallization process of PA6 and its nanocomposites [74]. The results obtained from this model are in accordance with the DSC analysis, described in Section 3.2 and the t_1/2_ parameter, described in the previous section.

#### 3.3.3. Friedman Model

Friedman [47] and Vyazovkin [75] developed an isoconversional method to calculate the E_c_ of the non-isothermal crystallization process. This method can be used to evaluate the dependence of E_c_ on crystallinity and temperature. From Equation (10), different activation energies can be calculated for each X_t_:

(10) $$ln\;{\left(\frac{dX}{dt}\right)}_{X,\;i}=constant-\frac{E_{c}}{{RT}_{X,i}}$$

where ln (*d*X/*d*t)_x_ is the instantaneous crystallization rate as a function of time for a given value of X_t_, R is the universal gas constant, *i* corresponds to each applied heating rate, and T_x_ is the temperature for a given scanning rate *i*. E_c_ can be calculated from plotting ln(*d*X/*d*t)_x_ versus 1/T_x_.

**Figure 6:**
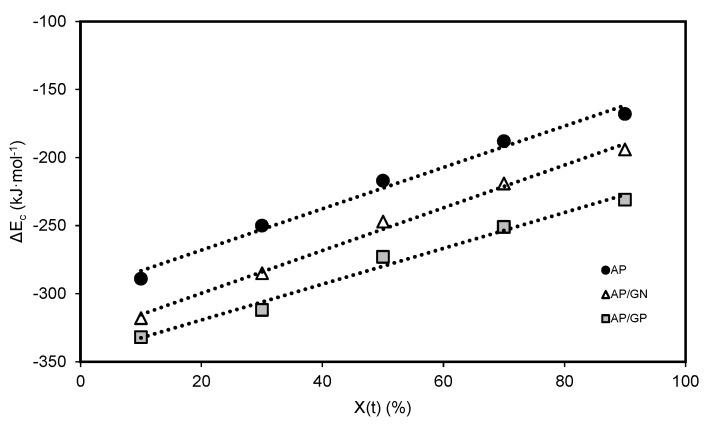
Friedman plots for PA6, PA6/GN, and PA6/GP.

Figure 6 shows the effective activation energy at a given crystallinity degree, and a regression coefficient of 0.99 was obtained for all samples.

As X_t_ increased, the values of E_c_ also increased, which reflects the difficulty of PA6 to crystallize as the crystallization process occurs [76]. Moreover, the E_c_ of PA6 decreased with the addition of GNPs. This is indicative that GNPs can act as nucleating agents to promote the initial stage of crystallization by inducing heterogeneous nucleation and, therefore, reducing the energy barrier. Comparing both nanocomposites, PA6/GN showed lower E_c_ than PA6/GP. These results are in accordance with those obtained previously for the Avrami and Liu models. Similar results have been reported [65,77].

## 4. Conclusions

The use of T-RTM technology was found to be successful for the preparation of PA6 and PA6/GNPs nanocomposites from AROP. The non-isothermal crystallization and kinetic behavior of developed PA6 and its nanocomposites were investigated. GNPs of two different sizes (GN and GP) were used. DSC trials were conducted at different cooling rates (5, 10, 15 and 20 °C/min). Results have shown that the incorporation of GNPs affected the crystallization behavior of PA6. Both GNPs led to an increase of T_c_, when compared to PA6, revealing the nucleation activity of the GNPs. Avrami and Liu’s models were selected to study the non-isothermal crystallization kinetics of all samples. Both models successfully described the behavior of PA6 and PA6/GNPs nanocomposites. The addition of GNPs increased the crystallization rate of PA6 and mixed growth mechanisms were observed (*n* < 4 and *b* > 1). Comparing both nanocomposites, the one prepared with GN (smaller diameter) exhibited a faster crystallization rate. These results were supported by Friedman’s isoconversional method, where a lower E_c_ was obtained for PA6/GNPs nanocomposites. Overall, these results indicated that all the kinetic models validated the nucleating effect of GNPs. A lower energy is needed for crystallization to take place in the presence of GNPs, which is more evidence for nanocomposites prepared with GN particles. GNPs size had a significant effect on the crystallization performance and it is expected that this behavior will affect the mechanical behavior of the final parts. Further studies are needed to understand the influence of higher GNPs concentrations on the crystallization behavior of PA6.

## Figures and Tables

**Table 1:** Non-isothermal crystallization parameters of PA6 and its nanocomposites.

Sample	φ (°C/min)	T_m_ (°C)	T_c_ (°C)	X_c_ (%)
PA6	5	218.9 ± 0.1	180.7 ± 0.4	38.6 ± 4.1
	10	219.1 ± 0.2	175.4 ± 0.3	39.4 ± 3.0
	15	218.9 ± 0.1	170.9 ± 0.7	40.8 ± 3.9
	20	218.8 ± 0.3	166.3 ± 0.5	41.6 ± 2.3
PA6/GN	5	219.1 ± 0.0	185.8 ± 0.6	39.6 ± 3.8
	10	219.2 ± 0.1	180.1 ± 0.4	40.9 ± 3.5
	15	219.0 ± 0.1	177.9 ± 0.4	41.5 ± 2.7
	20	218.9 ± 0.2	172.3 ± 0.3	42.4 ± 1.8
PA6/GP	5	218.8 ± 0.3	182.2 ± 0.8	39.0 ± 3.1
	10	218.8 ± 0.5	177.4 ± 0.7	40.6 ± 4.3
	15	218.6 ± 0.2	173.8 ± 0.7	41.3 ± 4.1
	20	218.1 ± 0.4	168.7 ± 0.9	41.9 ± 3.2

**Table 2:** Half-time crystallization and Avrami kinetic parameters.

Sample	φ (°C/min)	t_1/2_ (min)	Avrami		
			*n*	Z_c_ (min^−1^)	R^2^
PA6	5	2.33 ± 0.04	4.92 ± 0.06	0.68 ± 0.04	0.98
	10	1.48 ± 0.08	5.32 ± 0.12	0.89 ± 0.06	0.98
	15	1.04 ± 0.04	4.84 ± 0.09	0.98 ± 0.01	0.98
	20	0.84 ± 0.02	4.73 ± 0.04	0.99 ± 0.01	0.99
PA6/GN	5	2.01 ± 0.04	4.66 ± 0.07	0.82 ± 0.02	0.98
	10	1.20 ± 0.03	4.71 ± 0.05	0.92 ± 0.03	0.97
	15	0.95 ± 0.03	4.52 ± 0.05	1.01 ± 0.01	0.97
	20	0.67 ± 0.02	4.37 ± 0.04	1.02 ± 0.01	0.97
PA6/GP	5	2.04 ± 0.07	4.28 ± 0.10	0.73 ± 0.03	0.98
	10	1.38 ± 0.04	4.09 ± 0.09	0.90 ± 0.03	0.98
	15	1.14 ± 0.03	3.97 ± 0.04	0.97 ± 0.01	0.99
	20	0.86 ± 0.03	3.81 ± 0.03	1.00 ± 0.02	0.99

**Table 3:** Liu kinetic parameters.

Sample	φ (°C/min)	Liu			
		Xt (%)	*b*	F(T)	R^2^
PA6	5	20	1.31 ± 0.03	11.80 ± 0.11	0.99
	10	40	1.37 ± 0.01	14.58 ± 0.09	1.00
	15	60	1.37 ± 0.02	17.43 ± 0.14	1.00
	20	80	1.40 ± 0.03	22.93 ± 0.12	1.00
PA6/GN	5	20	1.43 ± 0.02	10.93 ± 0.16	0.99
	10	40	1.42 ± 0.02	13.18 ± 0.11	1.00
	15	60	1.46 ± 0.03	16.98 ± 0.17	1.00
	20	80	1.49 ± 0.01	21.91 ± 0.10	1.00
PA6/GP	5	20	1.47 ± 0.03	11.47 ± 0.21	0.99
	10	40	1.49 ± 0.02	14.01 ± 0.11	0.99
	15	60	1.51 ± 0.01	18.09 ± 0.12	0.99
	20	80	1.59 ± 0.02	21.52 ± 0.13	0.99

## Data Availability

The data presented in this study are available upon request from the corresponding authors.
